# The impact of diabetology consultations on length of stay in hospitalized patients with diabetes

**DOI:** 10.1002/edm2.199

**Published:** 2020-11-20

**Authors:** Kelsey H. Sheahan, Adam Atherly, Caitlyn Dayman, Joel Schnure

**Affiliations:** ^1^ Division of Endocrinology and Diabetes Larner College of Medicine at The University of Vermont Burlington VT USA; ^2^ Center for Health Services Research Larner College of Medicine at The University of Vermont Burlington VT USA

**Keywords:** cohort study, cost‐effectiveness, health economics

## Abstract

**Introduction:**

Both hyperglycaemia and hypoglycaemia in hospitalized patients have been shown to be associated with a longer length of stay, higher readmission rates, and higher rates of morbidity and mortality. With 25%‐30% of all hospitalized patients carrying a diagnosis of diabetes, it is important to optimize glycaemic control. Current guidelines for care of inpatients with diabetes now suggest consulting a specialized diabetes team for all patients when possible.

**Aim:**

This study was a retrospective cohort study to evaluate the impact of an inpatient diabetology consult within 48 hours of admission on patients’ length of stay.

**Methods:**

All patients admitted to the general medicine service between 2013 and 2018 with a diagnosis of diabetes in their medical record were included, which consisted of 11 477 inpatient stays. We looked at the effect of an inpatient diabetology consultation within the first 48 hours on length of stay, complications and 30‐day readmission rates.

**Results:**

We found that patients whose care included a diabetology consult within 48 hours of admission had a statistically significant shorter length of stay by 1.56 days compared to the remainder of the group. There was no difference in complications or 30‐day readmission rates between the groups.

**Conclusion:**

Among general medicine patients with a diagnosis of diabetes, timely diabetology consultations reduced patients’ length of stay and have the potential to improve their care and lessen the economic impact.

## Abstract 2

This is a retrospective cohort study of 11 477 patients over 5 years at an academic institution that examined the impact of an inpatient endocrinology consult for patients with diabetes on their length of stay. We found a statistically significant shorter length of stay by 1.56 days among patients who received an inpatient endocrinology consult within 48 hours of admission compared to the remainder of the patients.

## 1 INTRODUCTION

It has been shown that both hypoglycaemia and hyperglycaemia in hospitalized patients are associated with many adverse patient outcomes, including increased length of stay (LOS), higher rates of morbidity and mortality, and higher readmission rates.^1^, ^2^ Given that 25 to 30% of hospitalized patients have a diagnosis of diabetes, it is important to optimize diabetes care in order to diminish patient morbidity and mortality, and lessen the economic burden.^3^ In 2017, the estimated national cost of diabetes was $327 billion with an estimated 40.3 million inpatient days accrued by those with diabetes.^4^

An increasing number of studies show that consultations with specialized diabetes teams among hospitalized patients with diabetes resulted in a lower LOS, although these studies have all had fairly small patient populations.^5^, ^6^, ^7^, ^8^ The effects of specialized diabetes teams on readmission rates, however, are less clear. One study in Spain found no difference in readmission rates with or without inclusion of a specialized diabetes team in the patients’ care.^8^ On the other hand, a retrospective study comparing hospitalized patients cared for by a specialized diabetes team had significantly lower readmission rates when compared to patients cared for by a primary service team only.^5^ Further, another retrospective study showed a reduction in composite morbidity among hospitalized patients with diabetes if a consultation by a specialized diabetes team was provided.^9^ Given the limited evidence and the importance of diabetes care, a call to action was published in 2013 that outlined the need for further studies to evaluate morbidity, mortality, glycaemic control, and other outcomes in this patient population.^3^ However, due to the increasing evidence of the effectiveness of utilizing dedicated diabetes teams, current guidelines from the American Diabetes Association (ADA) recommend consulting a specialized diabetes team for hospitalized patients with diabetes when possible.^13^

Our aim was to look at a much larger patient population and to evaluate the impact of a diabetology consultation on LOS in patients with diabetes who were admitted to the general medicine service.

## 2 METHODS

Our study was a retrospective analysis of an electronic medical record (EMR) from a large hospital in the northeastern United States, which is an academic teaching institution, including an Internal Medicine residency programme and an Endocrinology, Diabetes and Metabolism fellowship programme. We collected hospital records for all stays between 2013 and 2018 for inpatients who were at least 18 years of age with either a diagnosis of diabetes in the patient's medical record or a diagnosis of diabetes from one of the hospital network's primary care professional sites 12 months prior to the inpatient event on the general internal medicine service. Patients admitted to the general medicine service are primarily cared for by a teaching team consisting of an attending physician, a resident, an intern and medical students, although a smaller subset of patients may be admitted and cared for by an advanced practice provider. Inpatient diabetology consultations (IDCs) are able to be called at any time, and patients are typically seen by an Endocrinology, Diabetes and Metabolism fellow and a supervising attending physician. Inpatient stays for pregnant women were excluded from the sample (n = 21 007). The sample was further limited to those patients with a stay of more than 48 hours (n = 2933) because our key independent variable was IDCs within the first 48 hours. We also excluded those with at least 1 day in the intensive care unit (n = 1383) given that our institution typically employs protocolized insulin infusions for most critically ill individuals, which does not require an IDC. These exclusions resulted in a final sample of 11 477 inpatient hospital events.

We utilized the EMR to access information about demographics, severity of illness (SOI), risk of mortality (ROM), readmissions and information about consultations with a diabetologist. We used the time stamp of the diabetology consult note creation to calculate the time from admission. In order to evaluate and control for severity of diabetes in this analysis, we calculated an average point of care blood glucose test (POC BG) based on those reported during the inpatient stay. Since the number of POC BGs varied, our average POC BG calculation used data from up to the first 10 POC BGs only.

We measured the effect of IDCs within the first 48 hours of admission on: (a) total LOS, measured in days from admission to discharge from the hospital; (b) complications, measured by unexpected inpatient events and (c) 30‐day all cause readmissions. We also measured the effect of IDCs at any point during patients’ hospitalizations on these outcomes. We controlled for patient characteristics: age, gender, SOI, ROM, BMI, average POC BG and any diabetology consultation prior to admission to the inpatient medical unit. LOS equations were estimated using a Poisson model while the probability of complications and the probability of readmissions were estimated using Probit models; for the Poisson models, the coefficients represent changes in days, while the Probit coefficients are marginal probabilities. All analyses were performed using Stata 15.

## 3 RESULTS

Only 3% of the hospital stays we examined had an IDC within 48 hours of hospital admission (Table 1). Those inpatient stays that received an IDC tended to be for patients that were younger or had higher average POC BG scores. Further, only 7.42% of the hospital stays received an IDC at any time during their hospital admission (n = 825).

**Table 1:** Descriptive statistics of inpatient hospital stays with general internal medicine among a diabetic population between 2013 and 2018 (N = 11 477)

	Did not receive consult in first 48 h (n = 11 064)	Received consult in 48 h (n = 413)
	**Mean (Standard Deviation)**	**Mean (Standard Deviation)**
Length of Stay (d)	6.94 (10.54)	6.19 (5.68)
Age (y)	67.51 (15.05)	55.93 (15.86)
Average POC BG (mg/dL)	174.66 (55.52)	211.97 (74.25)
BMI (kg/m^2^)	32.99 (76.19)	31.24 (9.36)
	**Per cent**	**Per cent**
Gender		
Male	54%	55%
Female	46%	45%
Severity of Illness at Admission		
Level 1	6%	7%
Level 2	32%	38%
Level 3	49%	46%
Level 4	13%	9%
Risk of Mortality at Admission		
Level 1	15%	22%
Level 2	37%	43%
Level 3	38%	30%
Level 4	11%	5%
Consultation prior to admission	0%	6%
Complications during admission	4%	3%
Readmission (related cause)	6%	9%
Readmission (all cause)	16%	14%

Table 2 displays the results of the Poisson regression analysing LOS. When compared to the mean LOS for all inpatient stays reviewed in this study, an IDC within 48 hours of admission led to a 1.56 day shorter average LOS that was statistically significant (*P* < .001). LOS was reduced by nearly half a day for females compared to males (*P* = .043) and decreased as individuals aged (*P* < .001). As the level of SOI and ROM increased, LOS is significantly longer. Average POC BG values, BMI and having a consult prior to hospital admission did not significantly change LOS for patients with diabetes in the general internal medicine unit.

**Table 2:** Poisson regression evaluating the change in length of stay (LOS) for diabetic patients during an inpatient stay on a general internal medicine unit

	Change in LOS (d)	Standard Error	95% Confidence Limits		*P* value
			Lower CL	Upper CL	
Diabetology Consult in 48 h	−1.56	0.38	−2.30	−0.82	.000
Age (per year older)	−0.09	0.01	−0.10	−0.07	.000
Female gender	−0.43	0.21	−0.84	−0.01	.043
Severity of Illness at Admission					
Level 2	0.70	0.23	0.26	1.15	.002
Level 3	3.25	0.30	2.67	3.84	.000
Level 4	6.38	0.51	5.38	7.37	.000
Risk of Mortality at Admission					
Level 2	1.30	0.30	0.72	1.89	.000
Level 3	1.61	0.36	0.91	2.30	.000
Level 4	2.12	0.49	1.15	3.09	.000
Average POC BG	0.00	0.00	−0.01	0.00	.460
BMI	0.00	0.00	0.00	0.00	.425
Consultation prior to admission	0.43	1.34	−2.20	3.07	.747

Table 3 shows that IDCs had no statistically significant effect on the rate of inpatient complications (*P* = .167). Female patients had a 1.0% lower chance of complications compared to males (*P* = .017). Patients with level 4 SOI at admission were 4.2% more likely to experience complications during the hospital stay compared to those admitted with a SOI at level 1 (*P* = .002); SOI levels 2 and 3 were not statistically significant. There was a significant increase in the likelihood of complications for each ROM level increase. Patients admitted with an ROM equal to 4 experienced a 3.4% higher rate of complications compared to patients with a ROM equal to level 1 (*P* = .001). The likelihood of complications increased as a patient's average POC BG value increased (*P* = .028). BMI, age and diabetology consultation prior to the hospital admission did not significantly impact a patient's likelihood of experiencing complications.

**Table 3:** Marginal effects of complications during an inpatient stay among a diabetic population on the general internal medicine between 2013 and 2018

	% point change	Standard error	95% Confidence Limits		*P* value
			Lower CL	Upper CL	
Diabetology Consult in 48 h	−1.7%	1.2%	−4.0%	0.7%	.167
Age (per year older)	0.0%	0.0%	0.0%	0.0%	.150
Female gender	−1.0%	0.4%	−1.7%	−0.2%	.017
Severity of Illness at Admission					
Level 2	−0.2%	0.9%	−1.9%	1.5%	.838
Level 3	1.4%	0.9%	−0.5%	3.2%	.145
Level 4	4.2%	1.3%	1.6%	6.8%	.002
Risk of Mortality at Admission					
Level 2	1.4%	0.6%	0.3%	2.6%	.015
Level 3	1.9%	0.7%	0.6%	3.2%	.004
Level 4	3.4%	1.0%	1.3%	5.5%	.001
Average POC BG	0.0%	0.0%	0.0%	0.0%	.028
BMI	0.0%	0.0%	0.0%	0.0%	.777
Consultation prior to admission	2.7%	4.1%	−5.4%	10.7%	.516

The probability of a readmission decreased for those with IDCs within 48 hours of admission, but not statistically significantly (Table 4). The probability of any readmission increased for those with an ROM at level 2 by 5.8% and by nearly 7.7% compared to those at level 1 (*P* < .001 and *P* < .001, respectively). The probability of readmission was 1.6% higher for women when compared to men (*P* = .025). SOI level, average POCT value, BMI and diabetology consults prior to admission did not significantly change the probability of readmission.

**Table 4:** Marginal effects of all cause readmissions after an inpatient stay among patients with diabetes on the general internal medicine service between 2013 and 2018

	% point change	Standard Error	95% Confidence Limits		*P* value
			Lower CL	Upper CL	
Diabetology Consult in 48 h	−2.9%	2.0%	−6.7%	1.0%	.144
Age (y)	−0.2%	0.0%	−0.2%	−0.1%	.000
Female gender	1.6%	0.7%	0.2%	3.0%	.025
Severity of Illness at Admission					
Level 2	1.8%	1.7%	−1.5%	5.2%	.279
Level 3	3.2%	1.8%	−0.3%	6.7%	.073
Level 4	2.7%	2.2%	−1.5%	7.0%	.212
Risk of Mortality at Admission					
Level 2	5.8%	1.0%	3.7%	7.8%	.000
Level 3	7.7%	1.3%	5.3%	10.2%	.000
Level 4	3.2%	1.7%	−0.1%	6.5%	.055
Average POC BG	0.0%	0.0%	0.0%	0.0%	.383
BMI	0.0%	0.0%	0.0%	0.0%	.337
Consultation prior to admission	0.4%	8.0%	−15.3%	16.1%	.959

Patients who received an IDC at any time during their hospitalization compared to those who never received a consult had a significantly longer LOS by 3.16 days (*P* < .0001). The rate of complications was also significantly increased by 2.2% in those who received an IDC consult at any time (*P* < .0001). The probability of readmission for those with IDCs at any time during their hospitalization was increased by 0.29%, although this was not significant (*P* = .731).

## 4 DISCUSSION

This study showed that among hospitalized patients with diabetes admitted to the internal medicine service, a consultation with diabetology within the first 48 hours of admission reduced the average LOS by 1.56 days. This reduced LOS was only significant if the IDC occurred within the first 48 hours of admission, with a significantly increased LOS among patients who received a consult at any time during their hospitalization, suggesting an optimal time for the initial consultation. Patients who received an IDC within 48 hours also had a slightly lower readmission rate, although this was not statistically significant.

With increasing evidence on the effectiveness of specialized diabetes teams in the hospital setting,^5^, ^6^, ^7^, ^8^ the current ADA guidelines recommend a consultation with a specialized diabetes team for all hospitalized patients with diabetes. Prompt IDCs during patients’ hospitalizations are thought to allow for better inpatient glycaemic control and education earlier in the hospitalization to prevent dysglycaemia and complications. This concept is not new, with a similar, although older, retrospective study showing that the effectiveness of a diabetes care team in reducing LOS was time dependent, with the earliest IDCs time receiving the largest reduction in LOS.^7^ IDCs later in patients’ hospitalizations often occur in more ill patients or as a result of complications, which could explain why patients who received an IDC at any time during their hospitalization had both a significantly increased LOS and rate of complications.

In addition to the positive impact of this reduction in LOS on a patient's quality of life, the economic impact of this reduction in LOS needs to also be considered. One of the largest contributors to the overall cost of diabetes is this population's higher utilization of inpatient services, which was estimated to cost $69.7 billion in 2017 with 40.3 million inpatient days.^4^ Specifically, at our institution, the average total medical charges per day among medicine patients is $6418. The average LOS was reduced by 1.56 days, which could mean a reduction in medical charges of more than $10 000 per patient stay at our institution. This knowledge suggests an important opportunity to reduce the economic burden of diabetes.

The 30‐day readmission rate has become an important metric of quality healthcare. The Centers for Medicare and Medicaid Services established the Hospital Readmission Reduction Program (HRRP), which looks at 30‐day readmission rates as a surrogate of quality healthcare and reduces payments to hospitals if they have high readmission rates.^10^ Interestingly, hospital readmission rates have significantly decreased since the implementation of HRRP, although it is unclear of the specific mechanism.^11^ Further, it is known that patients with diabetes have higher 30‐day readmission rates when compared to patients without diabetes, although there has been no defined intervention to date that has been able to improve this phenomenon. One study found a slightly lower readmission rate among those who received a consult within the first 48 hours although it was not statistically significant.^12^ 30‐day readmission rates remain an important outcome that needs further studies to determine ways to reduce this among patients with diabetes.

To our knowledge, this is the largest study to examine the effectiveness of an IDC on LOS in hospitalized patients with diabetes; however, it has several limitations. Our study is a retrospective analysis performed at one academic institution that cares for the population of Vermont and Northern New York and the results may not be generalizable to other hospital systems and populations. Additionally, we only looked at patients admitted to the general medicine service, which does not take into account the complexity and variety of medical conditions of patients on surgery and other services. Future aims are to look at these other services, as well as subpopulations of patients, including those receiving tube feeds or total parenteral nutrition, to evaluate the impact of IDC in these populations. Although patients receiving tube feeds or total parenteral nutrition were not excluded, the majority of these patients reside on surgical services which were not included; however, these patients require careful diabetes management given their changing nutritional status, making this an interesting patient population we hope to evaluate in further studies. Going forward, we also hope to obtain prospective data on IDCs and their impact not only on LOS but also complications, 30‐day readmission rates, and other important outcomes.

This study found that IDCs within the first 48 hours for patients with diabetes admitted to the general medicine service significantly reduced the LOS by 1.56 days. However, IDCs occurred in only a small minority of patients with diabetes, and less than half of the IDCs occurred within 48 hours of admission. This provides an important area to improve diabetes care in the hospital and potentially reduce economic costs.

## CONFLICTS OF INTEREST

The authors declare no conflicts of interest.

## AUTHOR’S CONTRIBUTION

KHS: study conception/design, data analysis, writing, editing and revising of manuscript. AA and CD: study conception/design, data acquisition, writing and editing of manuscript. JJS: study conception/design, data analysis, editing of manuscript.

## Data Availability

Our data are not available for sharing given it contains confidential patient information, but is stored on a secure server should access be needed.
